# Novel insights into the molecular mechanisms of LGMDD2: role of TNPO3 in experimental cell and zebrafish models

**DOI:** 10.1007/s00018-025-05954-9

**Published:** 2025-11-26

**Authors:** MT Rodia, M. Fazzina, Roberta Costa, MT Altieri, G. Sabbioni, E. D’Aversa, G. Breveglieri, E. Gatto, C. Bertolucci, S. Lombardi, M. Bergonzoni, R. Casadei, S. Santi, V. Papa, S. Ratti, G. Cenacchi, M. Borgatti, F. Frabetti

**Affiliations:** 1 Cellular Signaling Laboratory, Anatomy Center, Department of Biomedical and Neuromotor Sciences, Alma Mater Studiorum University of Bologna, Bologna, Italy; 2 Department of Pharmacy and Biotechnology, Alma Mater Studiorum University of Bologna, Bologna, Italy; 3 Department of Life Sciences and Biotechnology, University of Ferrara, Ferrara, Italy; 4 Department of Translational Medicine, University of Ferrara, Ferrara, Italy; 5 Department of Chemical, Pharmaceutical and Agricultural Sciences, University of Ferrara, Ferrara, Italy; 6 The Wistar Institute, Philadelphia, PA USA; 7 Department for Life Quality Studies, Alma Mater Studiorum University of Bologna, Bologna, Italy; 8 Institute of Molecular Genetics “Luigi Luca Cavalli-Sforza”, Unit of Bologna, CNR—National Research Council of Italy, Bologna, Italy; 9 IRCCS Istituto Ortopedico Rizzoli, Bologna, Italy; 10 UOC Anatomia ed Istologia Patologica, IRCCS Policlinico Sant’Orsola Malpighi, Bologna, Italy; 11 Department of Medical and Surgical Sciences, Alma Mater Studiorum University of Bologna, Bologna, Italy

**Keywords:** LGMDD2, Transportin 3, Myogenesis, C2C12, Zebrafish, MyomiRNA

## Abstract

Transportin 3 (TNPO3) is a nuclear carrier for serine/arginine-rich proteins which are essential for mRNA splicing and metabolism. Mutations in the *TNPO3* gene result in a protein with an extended C-terminal domain, leading to the onset of LGMDD2, a rare form of limb girdle muscular dystrophy. To investigate the role of TNPO3 in skeletal muscle and the pathogenic mechanism underlying LGMDD2, we develped both in vitro and in vivo models of the disease. The in vitro model was established using the C2C12 cell line, transfected with plasmids encoding either the wild-type (WT) or mutated (MUT) human *TNPO3* (hTNPO3). For the in vivo model, we microinjected zebrafish (*Danio rerio*) embryos with mRNAs encoding WT or MUT hTNPO3. We analyzed the expression patterns of myogenesis-related genes, muscle-specific genes, myomiRNA and genes strictly related to the disease. These analyses were complemented by protein expression studies, morphological assessments in both models, and behavioral assays to validate the LGMDD2 zebrafish model. Our results demonstrate a key role for TNPO3 in regulating myogenesis in both models and reveal that the TNPO3 mutation disrupts normal myogenic commitment, supporting its contribution to LGMDD2 pathogenesis. Overall, this study represents a significant advance in understanding the role of TNPO3 in skeletal muscle biology and the molecular basis of LGMDD2.

**Supplementary Information:**

The online version contains supplementary material available at 10.1007/s00018-025-05954-9.

## Introduction

Transportin 3 (TNPO3), belonging to the beta karyopherin family, is a nuclear carrier for serine/arginine-rich (SR) proteins [1], which are essential for mRNA splicing and metabolism [2, 3]. Mutations in the *TNPO3* gene (OMIM 608423) have been associated with a rare form of limb-girdle muscular dystrophy: LGMDD2 (previously LGMD1F) or LGMDD2 TNPO3-related [4]. The first identified mutation is a heterozygous deletion of a single adenine nucleotide in the stop codon (c.2771delA); additional point mutations have also been described, all leading to a protein that is 15 aminoacids longer in its C-terminal domain compared to the wild-type [5–11].

Clinically, LGMDD2 is characterized by high clinical variability and, unlike other dystrophies and forms of LGMD marked by necrotic degeneration and regeneration, its main distinguishing feature is a generalized and pronounced muscle atrophy [12]. Recently, a proof-of-concept study demonstrated the successful use of CRISPR-Cas9 to correct the causative genetic defect and restore the wild-type TNPO3 sequence [13]. Despite these findings, including the identification of novel mutations and detailed characterization of clinical and histopathological features of LGMDD2, the role of TNPO3 in skeletal muscle and the underlying pathological mechanisms remain unclear. It has been proposed that TNPO3 may function as a dimer [14–16], and the heterozygous mutations could affect protein dimerization, stability, and/or function, leading to impaired nuclear import of splicing factors and proteins involved in RNA metabolism. On the other hand, the mutation in TNPO3 could lead to the formation of aggregates containing TNPO3 and its specific cargoes, such as the SRSF1-positive aggregates described in the muscle biopsies of patients [17]. Both mechanisms could directly disrupt the splicing machinery, reducing the proteomic variability in skeletal muscle and contributing to a defective myogenic process in the disease pathogenesis.

In this work, we created both in vitro and in vivo models of LGMDD2 to better understand the role of TNPO3 in the myogenic process and in its pathogenetic mechanism. Using the C2C12 cell line, a standard model for muscle commitment and disorders, we generated the in vitro model by transfecting a plasmid carrying either the wild-type (WT) or mutated (MUT) sequence of human *TNPO3* (*hTNPO3*) [18].

For the in vivo model, we employed the teleost zebrafish (*Danio rerio*), a prominent vertebrate model for translational research due to its low-cost maintenance, high fecundity, and the fast development of its easily manipulable embryos [19]. Myogenesis has some unique features in teleosts compared to mammals, which include the early stage of muscle commitment, different proportions of slow and fast fibers, and muscle growth throughout much of ontogeny. Neverthless, aspects referred to the physiogical development, genes and proteins involved in myogenic differentiation, that is direcly observable in the teleost, are highly conserved between humans and zebrafish [20]. Moreover the skeletal muscle is already functional as early as 20 hours post fertilization (hpf) and at 48 hpf the larva is able to swim spontaneously offering the opportunity to follow possible structural and functional alterations [21]. *Tnpo3* in zebrafish exists as a single gene copy and bioinformatic analysis has shown 86% homology of the protein sequence with its human counterpart (data not shown); all these characteristics make this teleost an ideal model to study the molecular mechanisms of LGMD subtypes [22]. Similar to the cell model, we microinjected mRNAs coding for WT or MUT *hTNPO3* in zebrafish embryos. To gain clearer insight into the molecular pathways involved in the disorder’s pathogenesis, we focused on alterations in the expression patterns of myogenesis-related genes, especially myogenic regulatory factors (MRFs) and muscle-specific genes. We further investigated myocyte enhancer factor 2 C (MEF2C) and its α1 and α2 isoforms, as SRSF1 and RBM4, two splicing factors transported by TNPO3, have been shown to regulate MEF2C splicing [23, 24]. Moreover, *MEF2* genes are highly expressed in murine muscle cells and play a major role in their development and differentiation, since MEF2 proteins functionally interact with members of the MRF family [25–27]. Furthermore, we analyzed the expression of some muscle-specific microRNAs, known as myomiRNAs or myomiRs (miR-1, miR-206, miR-133a and miR-133b), which are involved in myogenesis, myoblast differentiation and proliferation [27, 28]. Their expression is regulated by MRFs and MEF2 through feedback loops that play an active role during myogenesis [29–34]. Additionally, alterations in myomiRNAs, such as miR-206 [35], have been documented in LGMDD2 and several muscular dystrophies [35–38].

A detailed analysis of gene and myomiR expression related to myogenesis was followed by investigations of protein expression and morphological changes in cells and zebrafish embryos during this process, aiming to confirm the deregulation of myogenic commitment at the morphological level. Finally, the analysis has been extended to behavioral and functional tests to validate the LGMDD2 zebrafish model.

Our data suggest that TNPO3 mutation is linked to impaired myogenesis in both cell and zebrafish models, confirming that precise regulation of the developmental muscle structure is crucial for proper myogenic commitment and its deregulation plays a role in the pathogenic mechanism of LGMDD2.

## Materials and methods

### Plasmid DNA amplification and mRNA in vitro transcription

The plasmid pCMV6-AN-GFP, carrying the WT or MUT h*TNPO3* sequence (Origene Technologies), was used to transform One Shot^®^ TOP10 chemically competent *E.coli* (Life Technologies), and plasmid DNA was extracted using the QIAGEN Plasmid Kit.

The plasmid DNA was then digested and linearized for in vitro transcription with the mMESSAGE mMACHINE^®^ Kit (Invitrogen Life Technologies). The resulting WT or MUT mRNA was used for microinjection into zebrafish embryos.

### Cell cultures and plasmid transfection

Murine myoblasts C2C12 (RRID: CVCL_0188; ATCC) were cultured as previously described [14]. For transfection, 4 µg of pCMV6-AN-GFP carrying either WT or MUT h*TNPO3* were added to Ingenio electroporation solution (Mirus Bio), and the cells were electroporated using the appropriate nucleofection program. Neomycin-resistant cells were then placed in 96-well plates for clonal selection.

### C2C12 myogenic differentiation gene and protein analyses

The selected C2C12 clones carrying the pCMV6-AN-GFP WT h*TNPO3* (WT) or the pCMV6-AN-GFP MUT h*TNPO3* (MUT) plasmids were expanded and induced to myogenic commitment as previously described [14]. Non-transfected C2C12 were used as control (CTRL). Cell samples were collected at four differentiation time points: T0, T1, T5 and T10, representing days after the induction of differentiation.

RNA and protein extraction, purification and quantification were performed as previously described [14]. Real-time qPCR analysis was performed in triplicate to evaluate gene expression, with qPCR signals (Cq values) normalized to glyceraldehyde 3-phosphate dehydrogenase (*Gapdh*). All primers are listed in Online Resource 1. Total cell lysate proteins were quantified and analyzed by western blotting (WB) as previously described [14].

To evaluate the expression of markers of interest, 10.000 cells/cm^2^ were seeded on Nunc LabTek Chamber Slides (Thermo Fisher Scientific) followed by immunofluorescence (IF) staining and image acquisition using a confocal microscope, as described [14]. Antibodies used for IF and WB are listed in Online Resource 2.

### Animal care

Zebrafish (ZF) embryos were raised and maintained according to the European Legislation for the Protection of Animals used for Scientific Purposes (Directive 2010/63/EU) and the Italian animal protection standards (Italian decree 26/2014). The University of Bologna holds License number 270,250/2021 for fish maintenance and breeding. All experiments were performed on embryos within 5 days post fertilization (dpf). Although European and National directives do not require a permit for testing zebrafish embryos at early developmental stages (>5 dpf), we conducted all experiments in accordance with ARRIVE guidelines [39].

### Microinjection of zebrafish embryos

Fertilized eggs were collected through natural spawning in clean breeding tanks and then transferred to Petri dishes containing E3 medium [40]. One-cell stage zebrafish embryos were then collected for microinjection following the procedure described [41]. Fifty pg of h*TNPO3* WT or h*TNPO3*-MUT mRNA, along with phenol red (Sigma) as a tracer for microinjection efficacy, were injected. Non microinjected (NI) embryos served as controls. After microinjection, both NI and microinjected embryos were transferred back to Petri dishes containing fresh E3 medium and placed in an incubator at 28.5 °C. The embryos were regularly monitored for their development and general health and staged according to the reference guidelines [42].

### Zebrafish gene expression analyses

Pools of 20–25 whole zebrafish embryos at 24 and 48 hpf were manually homogenized in 750 µL of TRIzol^®^ (Invitrogen). Total RNA was extracted using a RNeasy Mini kit (Qiagen) and 1 µg of RNA was reverse transcribed with iScript RT Supermix (Bio-Rad). Real-time PCR reactions were performed in triplicate with SsoAdvanced Universal SYBR Green Supermix (Bio-Rad), and qPCR signals (Cq) were normalized to the expression levels of two housekeeping genes: *actb2* (*actin beta 2*) and *slc25a5* (*solute carrier family 25 member 5*), using the Livak (or ΔΔCq) method, as previously described [43]. Primers are listed in Online Resource 3.

### MicroRNA analyses on C2C12 and zebrafish samples

For miRNA analysis, total RNA was extracted from C2C12 cells (1 × 10^6^ cells) using the miRNeasy Mini Kit (Qiagen), according to the manufacturer’s instructions. Zebrafish embryos were homogenized with 700 µL of QIAzol Lysis reagent (Qiagen) for RNA extraction with the same kit.

Quantification of miR-206, miR-1, miR-133a and miR-133b was performed by qRT-PCR in a two-step process. First, 500 ng of each RNA sample were reverse transcribed using the TaqMan™ MicroRNA Reverse Transcription Kit (Applied Biosystems, Thermo Fisher Scientific). In the second step, cDNA was amplified by Real-Time PCR using the CFX96™ Touch Real-Time PCR Detection System (Bio-Rad Laboratories) and specific TaqMan™ MicroRNA Assays (Applied Biosystems). Normalization was conducted using U6 snRNA as housekeeping gene. The ∆∆Cq method [44] was employed to calculate the relative expression of miRNAs. In C2C12 analysis, miRNA expression was compared to control samples at the T0 stage, while in zebrafish analysis it was compared to NI samples at the corresponding developmental stage.

### Immunofluorescence analysis on zebrafish

Immunofluorescence assays were performed on both injected and NI zebrafish embryos at 48 hpf. Embryos were dechorionated using a solution of 2 mg/ml Pronase in E3 Medium, fixed in 4% paraformaldehyde (Alfa Aesar) and washed in PBST before pigmental removal (Pigmental removal solution composed by KOH 0.8%, H_2_O_2_ 0.9%, Tween 20 0.1% in H_2_O). After three washes in PBST, embryos were incubated in 0.1% Collagenase P (Roche) in HBSS at 37 °C for 30 min, washed in PBST and post-fixed in 4% paraformaldehyde with 0.4% Triton X. After three additional washes in PBST, non-specific binding sites were blocked by PBST containing 5% goat serum (Merck) for 1 h at room temperature with gentle rocking. Embryos were then incubated overnight at 4 °C with a specific monoclonal anti α-actinin (A7811, Merck) and anti skeletal muscle myosin (sc-32732, Santa Cruz) antibody in PBST with 1% goat serum. The next day, embryos were washed three times in PBST with gentle rocking and incubated overnight at 4 °C with a Cy™3 AffiniPure Goat Anti-Mouse IgG (H + L) secondary antibody (Jackson ImmunoResearch). Following three additional PBST washes, embryos were mounted in glass-bottom dishes (FluoroDish, WPI) and observed using a Nikon A1 confocal laser scanning microscope equipped with a 40x, 1.4 NA objective and with 560 nm laser lines.

### Ultrastructural investigation on zebrafish skeletal muscle by transmission electron microscopy (TEM)

Zebrafish embryos at 48 hpf and 4 dpf were fixed in 2.5% glutaraldehyde in 0.1 M cacodylate buffer (pH 7.4), post-fixed in 1% OsO_4_ in the same buffer, dehydrated and embedded in epoxy resins as previously described [6]. Thin sections were stained with uranyl acetate and lead citrate, then examined using a Philips TEM CM 100.

### Functional analysis of zebrafish larvae behaviour

At 5 dpf, behavioural responses were assessed in mutant and control groups (NI and phenol red microinjected, PR) in two consecutive tests using the DanioVision Observation Chamber (Noldus). In the first test, we investigated behavioural changes in a novel environment using the open-field test [45–47]. Responsive larvae were randomly selected and placed in a 24-well culture plate (5 replicates), with locomotory activity recorded for 60 min. EthovisionXT tracking software (ver. 11.5, Noldus) was used to quantify three parameters:

1. total distance moved (cm) across the arena (well Ø 19 mm);
2. swimming velocity (cm/s);
3. time spent in freezing (s), with settings: average interval = 5 samples, start velocity = 2.00 mm/s.

Larval size was also measured to assess developmental differences among groups. Five video frames were extracted per subject, and individual size were measured using ImageJ.

Following the open-field test, cognitive function was evaluated by exposing the larvae to repeated vibrational stimulations (habituation learning test) [48]. Mechanical stimulation was administered via a solenoid (Tapping Device, Noldus) controlled by EthovisionXT software. Each stimulus consisted in a mechanical vibration that elicited an escape response, observed as increased swimming activity (startle response). Habituation is generally measured by evaluating the decrease in the startle response through repeated stimulation [48–50]. Cognitive performance was assessed using three parameters:

4. distance moved (cm) by subject in response to the first stimulus;
5. total distance moved (cm) across the 25-repeated stimulations;
6. a habituation index based on the differences between the first stimulus and the following ones divided for the first stimulus.

Due to the right-skewed distribution of the data, distances were log-transformed before analysis. Seven larvae (3 NI, 1 PR, 3 MUT) remained immobile during the two tests and were excluded from the analysis. Final sample sizes were: MUT = 33, PR = 39, NI = 37.

### Statistical analyses

Gene expression and WB data from C2C12 cells were obtained from triplicate experiments. Similarly, miRNA analyses were also performed in triplicate. In zebrafish, gene expression analysis was conducted on pools of 20–25 embryos for each condition (NI, h*TNPO3* WT or h*TNPO3* MUT), with 5 to 9 biological replicates per group. WB analysis was performed on pools of 30 embryos for each condition, with three replicate for each group. Finally, miRNA analysis was conducted on pools of 25–30 embryos, with 3 to 7 replicates for each condition. Statistical analyses were performed using GraphPad Prism software, version 10.2.1 (GraphPad Software, Inc.). Student’s *t*-test or one-way ANOVA were used where appropriate. For C2C12 miRNA expression, Tukey’s Multiple Comparison test was applied. For zebrafish miRNA analysis, Brown-Forsythe and Welch ANOVA tests were used, followed by Tukey’s Multiple Comparison test.

## Results

### Myogenic differentiation of C2C12 cell model

Transfection of C2C12 cells with plasmids encoding either WT or MUT h*TNPO3* allows observation of the effects of TNPO3 overexpression and mimics the pathological condition LGMDD2. We investigated the myogenic differentiation of transfected C2C12, focusing on alterations in the expression patterns of MRFs, MEF2C and muscle-specific proteins.

**Fig. 1:**
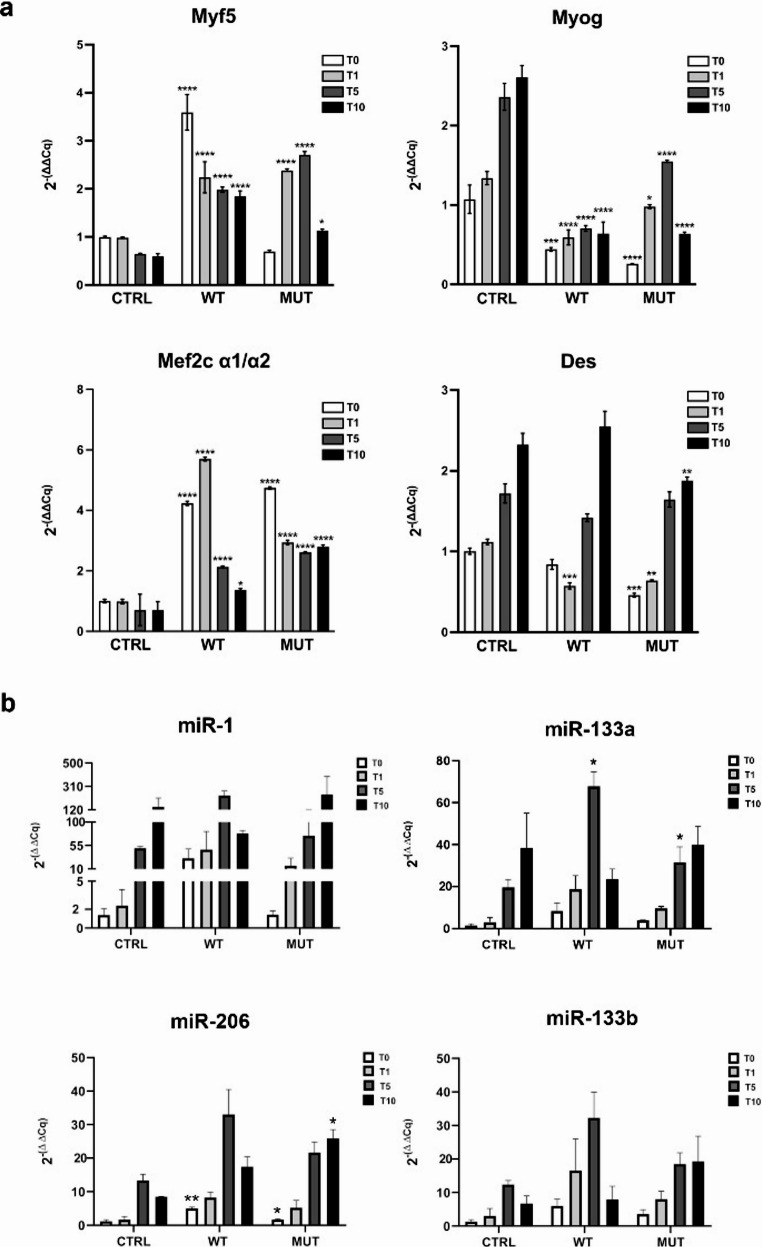
Graphical representation (mean values with SEM) of relative gene expression levels of *Myf5*, *MyoG*,* Mef2C α1/α2* ratio, *Desmin (Des)* (**a**) and of miR-1, miR-133a, miR-133b and miR-206 (**b**) in C2C12 cell model. In all graphs CTRL are non-transfected C2C12, WT and MUT are C2C12 transfected with plasmids carrying respectively WT or MUT h*TNPO3* sequence. Statistical analysis, panel a: one-way ANOVA and Bonferroni’s Multiple Comparison test (at least *n* = 3); panel b: one-way ANOVA and Tukey’s Multiple Comparison test were used; *p < 0.05; **p < 0.01; ***p < 0.001. The statistical significance depicted in the graphs demonstrates the comparative analysis among CTRL, WT, and MUT for each differentiation stage (T0, T1, T5 and T10)

The relative gene expression of *Myf5* increased in both WT and MUT h*TNPO3* transfected cells during differentiation, while *Myog* expression decreased compared to control cells (Fig. 1a). To better understand the full picture of regulatory pathways involving the MRFs, we analyzed *Mef2C* expression. As a member of the Mef2 protein family, *Mef2C* cooperates with MRFs and is regulated by them. Its primary transcript contains two mutually exclusive exons, *α1* and *α2*, which define the functional outcome: the α1 isoform maintains an undifferentiated state by recruiting HDAC5 (Histone Deacetylase 5) and repressing muscle-specific genes, while the α2 isoform promotes myoblast differentiation. Figure 1 shows the *α1/α2* ratio across conditions, suggesting that both WT and MUT h*TNPO3* support the undifferentiated state compared to controls. As differentiation progresses, this ratio decreases, consistent with the promotion of differentiation.

Interestingly, expression of the muscle-specific transcript *Desmin* was reduced in both MUT and WT h*TNPO3* transfected cells, particularly during the early phases of differentiation. MyomiRs affect the balance between differentiation and proliferation through interactions with multiple targets and their expression increases during C2C12 myoblast differentiation [51]. Our data support this, showing increased expression of miR-1, miR-133a, miR-133b and miR-206 in WT h*TNPO3* transfected cells at T0-T1 and T5, compared to not transfected controls and MUT h*TNPO3* transfected cells. The upregulation was statistically significant for miR-133a and miR-206 (Fig. 1b). On the other hand, in C2C12 cells transfected with MUT h*TNPO3* at T0-T1 and T5, all four myomiRs showed a downregulation trend, which appeared statistically significant for miR-133a at T5, if compared to WT h*TNPO3* transfected cells. At T10, gene expression levels of all the analysed myomiRs increased in MUT h*TNPO3* compared to WT and CTRL groups, with miR-206 showing a statistically significant increase.

### Expression of LGMDD2 specific genes and proteins

**Fig. 2:**
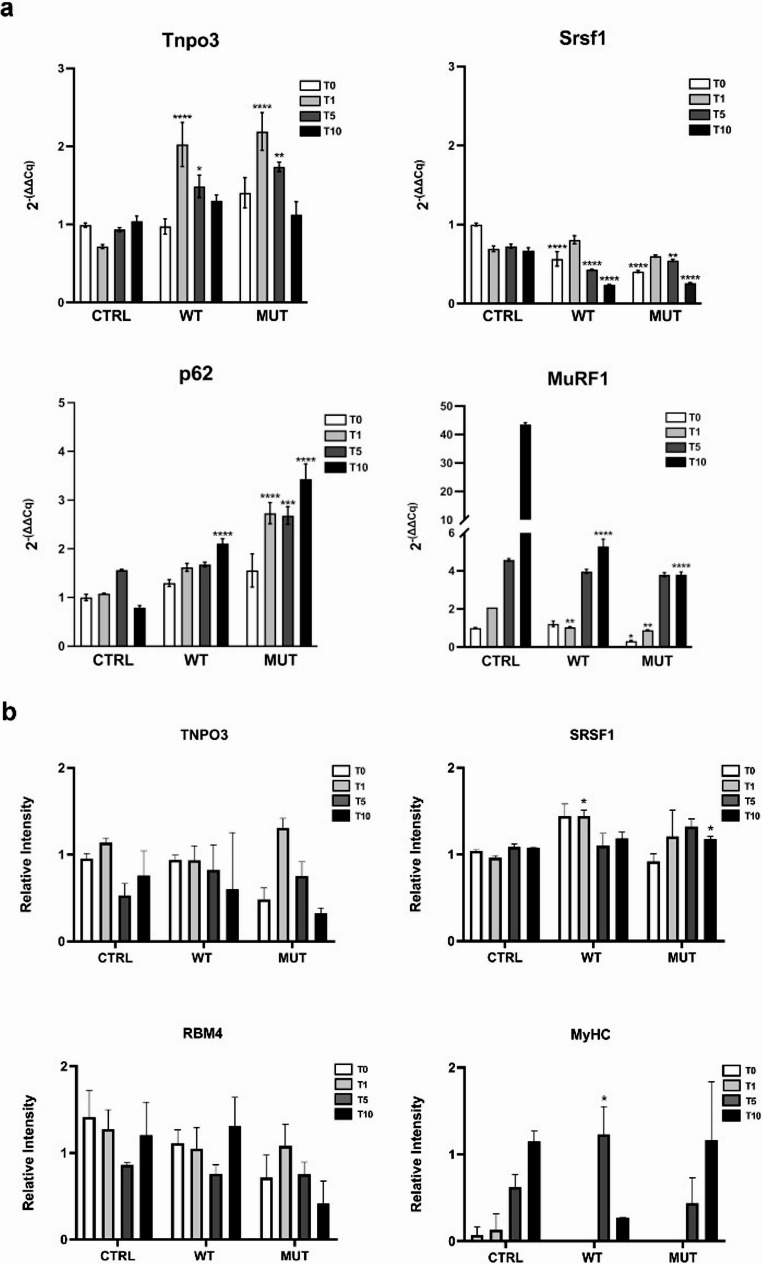
(**a**) Graphical representation of the relative gene expression levels of *Tnpo3*,* Srsf1*,* p62* and *MuRF-1.* (**b**) Western blot analysis of TNPO3, SRSF1, RBM4 and MyHC; the graphs show protein levels normalized to ACTB. In all graphs CTRL refers to non-transfected C2C12, while WT and MUT refer to C2C12 transfected with plasmids carrying the h*TNPO3* WT or MUT sequence, respectively, at different time points after the induction of differentiation: T0, T1, T5 and T10. The WB on MyHC showed no band for both WT and MUT at T0 and T1, and as a consequence no bar in the relative intensity graph. Statistical analysis: one-way Anova and Bonferroni’s Multiple Comparison test (at least *n* = 3); *p < 0.05 and statistical significance based on comparative analyses among CTRL, WT, and MUT for each time-point

The expression of *Tnpo3* was studied during myogenic differentiation. A statistically significant increase at T1, T5, and T10 was observed in cells transfected with WT or MUT h*TNPO3* compared to non-transfected controls (Fig. 2a). We also evaluated the expression of *Srsf1*, a known TNPO3 cargo. Its expression profile was similar to that observed for *Tnpo3*, but showed lower levels in transfected cells. To better characterize the cell model, we analyzed *p62* and *MuRF-1*, markers of autophagy and muscle atrophy, respectively. We focused on these since marked atrophy is a hallmark of LGMDD2 [6], and the only Drosophila model developed to date showed a direct link between LGMDD2 and upregulated autophagy [52]. Our results showed a significant increase in *p62* expression in both WT and MUT h*TNPO3* transfected cells at all differentiation stages. In contrast, *MuRF-1* expression, which increased over time in CTRL samples, was dramatically reduced in transfected cells (Fig. 2a).

We also performed WB to analyse the main gene products involved in muscle differentiation. Although we could not distinguish between endogenous and human proteins, we noticed slight variations in TNPO3 protein and its cargos (SRSF1 and RBM4) levels during differentiation. Notably, SRSF1 showed a significant increase at T1 in WT and at T10 in MUT transfected cells compared to controls, suggesting that its expression is affected by TNPO3 overexpression or by the presence of human mutant protein. Additionally, myosin (Myosin Heavy Chain, MyHC) expression significantly increased at T5 in WT h*TNPO3* cells, confirming that h*TNPO3* transfection can affect the expression of muscle-specific proteins (Fig. 2b).

**Fig. 3:**
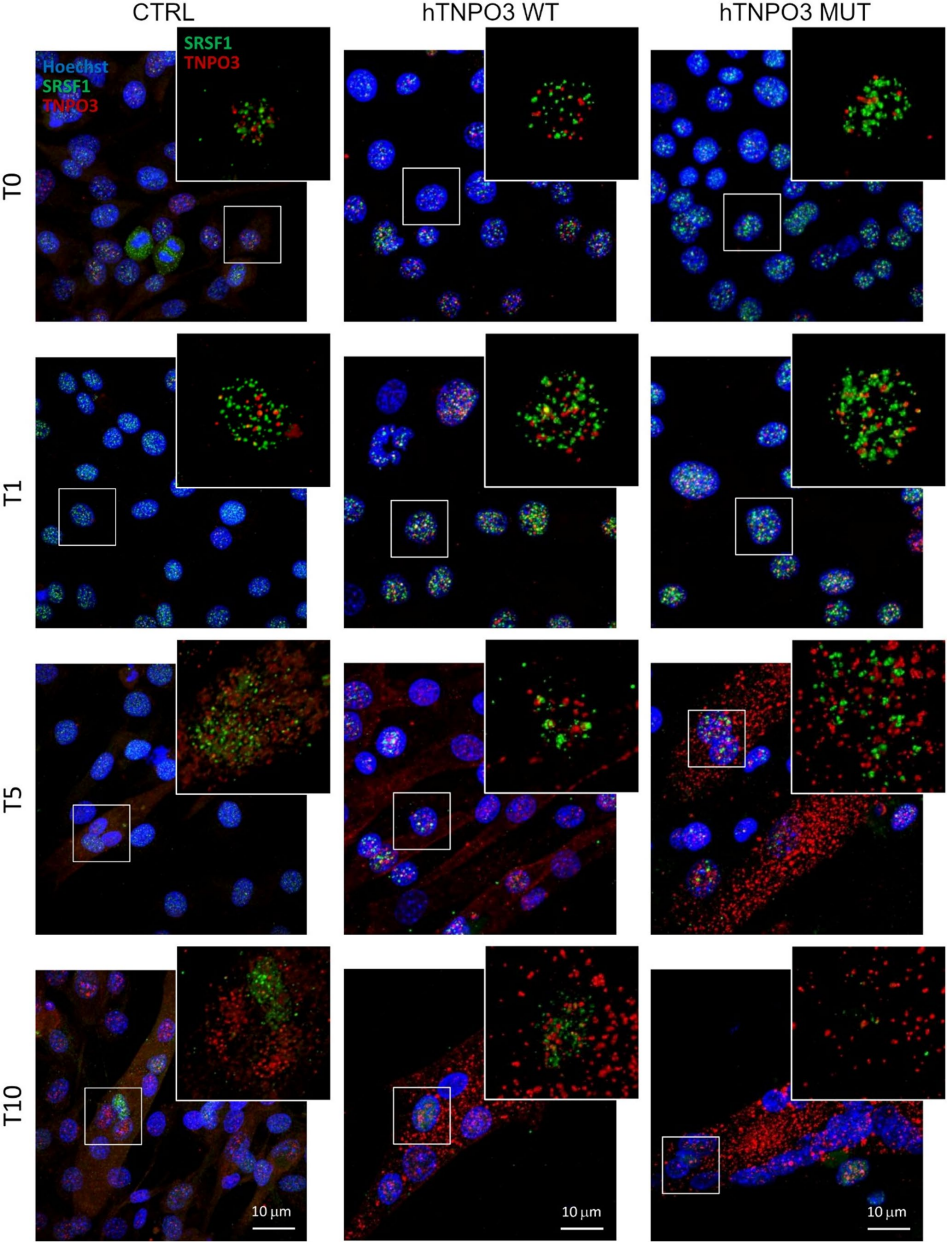
Analysis of TNPO3 and SRSF1 localization during myogenesis by confocal microscopy. TNPO3 in red, SRSF1 in green and nuclei are counterstained with Hoechst in blue. CTRL are non-transfected C2C12, while h*TNPO3* WT or MUT are C2C12 transfected with plasmids carrying WT or MUT sequence at different times after differentiation induction: T0, T1, T5 and T10. Scale bar 10 μm

Double immunofluorescence stainings for TNPO3 and SRSF1 and for TNPO3 and myosin confirmed the previously described behavior of TNPO3 in C2C12 [14], which localizes mainly in the cytoplasm of myoblasts that respond to the myogenic stimuli. Figure 3 showed no marked difference in TNPO3 staining between control and transfected cells. Regarding SRSF1, staining is mostly limited to the nuclei throughout differentiation. Of note, cells transfected with h*TNPO3* WT or MUT showed larger aggregates positive for SRSF1 from T0 to T5, but at T10 the size and number of SRSF1 aggregates is clearly reduced.

**Fig. 4:**
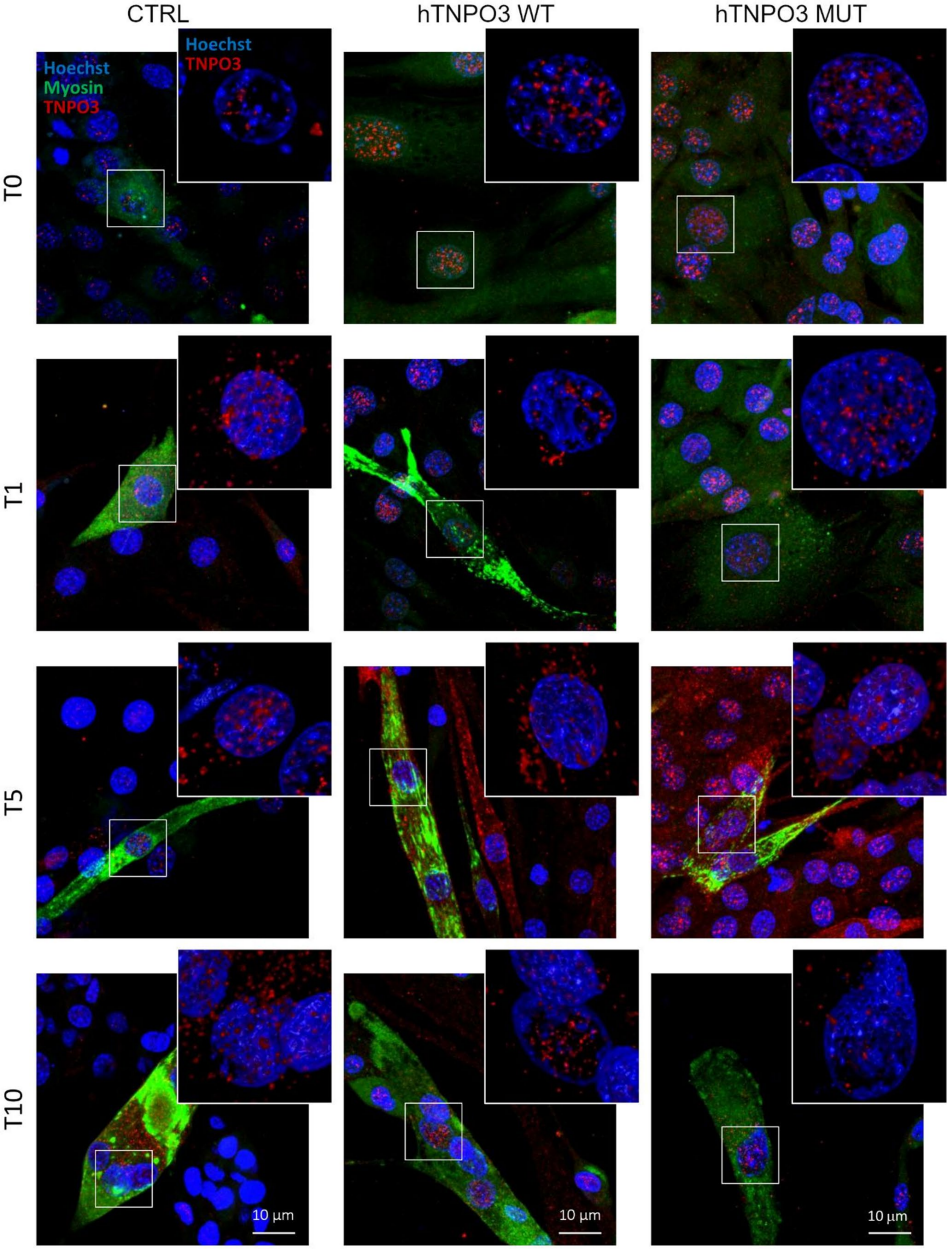
Analysis of TNPO3 and myosin during myogenesis by confocal microscopy. TNPO3 in red, myosin in green, nuclei in blue. CTRL are non-transfected C2C12, while hTNPO3 WT or MUT are C2C12 transfected with plasmids carrying WT or MUT sequence at different times after differentiation induction: T0, T1, T5 and T10. Scale bar 10 μm

In Fig. 4, myosin starts to be expressed and well organized in the cytoplasms of differentiating myoblasts from T1, with the appearance of multinucleated and elongated myotubes at T10 in both non transfected and transfected with h*TNPO3* WT cells. In contrast, cells transfected with hTNPO3 MUT at T10 display shrunken myotubes that are positive for myosin but show a reduced number of nuclei and enlarged, rounded edges.

### Generation of the zebrafish model of LGMDD2 and evaluation of MRFs and muscle-specific genes

**Fig. 5:**
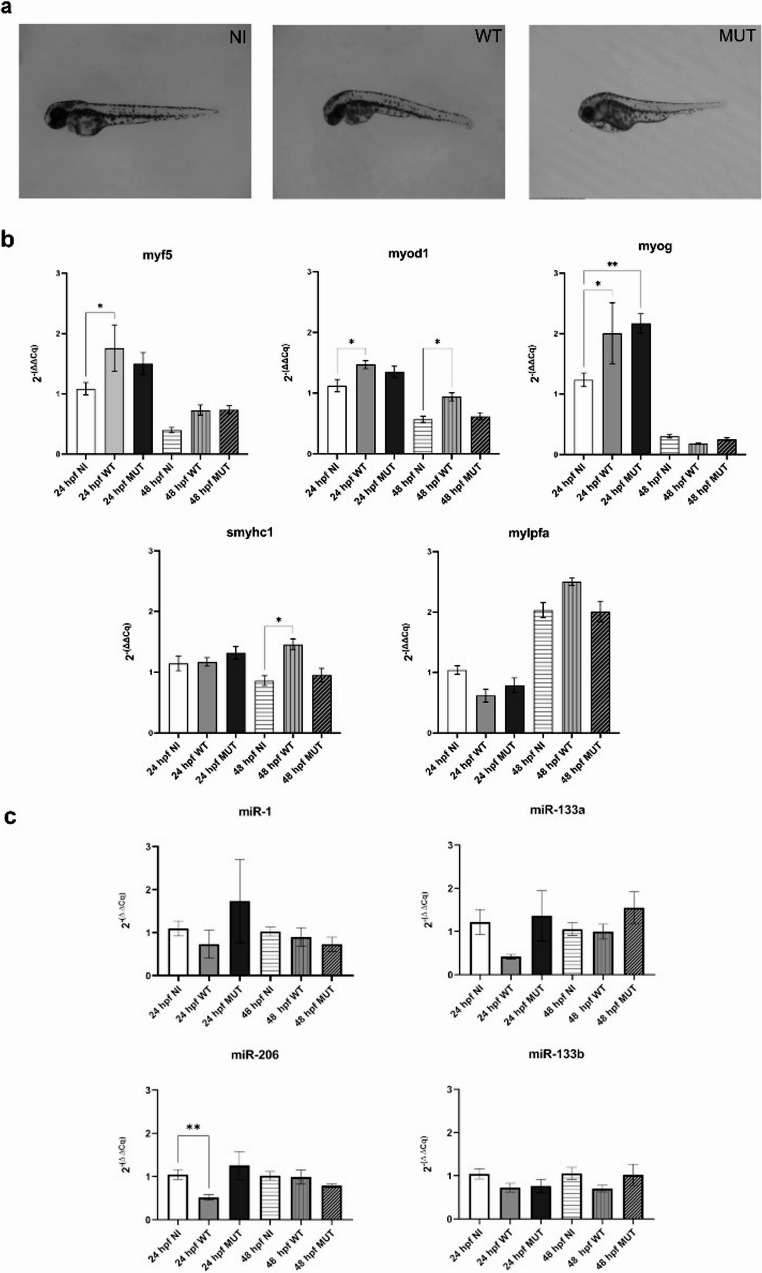
(**a**) Morphology of zebrafish embryos NI and microinjected with 50 pg of WT or MUT h*TNPO3* mRNA at 48 hpf. Graphical representation (mean ± SEM) of relative gene expression levels of (**b**) *myf5*,* myod1*,* myog*,* mylpfa* and *smyhc1* and (**c**) miR-1, miR-133a, miR-133b and miR-206. All data refer to NI or microinjected zebrafish embryos at 24 hpf and 48 hpf (minimum *n* = 5). For miRs analysis, Welch and Brown-Forsythe ANOVA with Tukey’s Multiple Comparison test were used. *p < 0.05; **p < 0.01. Statistical significance depicted in the graphs demonstrates the comparative analysis among CTRL, WT and MUT at each time point (24 or 48 hpf)

We identified 50 pg as the optimal amount of mRNA for microinjection, which resulted in reduced somite size (data not shown) and overall body shortening in embryos microinjected with h*TNPO3* MUT mRNA (Fig. 5a).

As with the C2C12 model, we focused on possible alterations in the expression of MRFs and muscle-specific genes. The expression levels in NI samples were consistent with the literature for each transcription factor considered, while the microinjection of either h*TNPO3* WT or MUT mRNA led to altered expression of *myf5* and *myod1*, which are early MRF genes typically activated at the onset of myogenesis and downregulated thereafter, as well as *myog*, that is a MRF factor activated later in myogenesis. Notably, h*TNPO3* WT microinjected embryos showed a significant increase in *myf5* and *myod1* expression at 24 and 48 hpf if compared to NI embryos (Fig. 5b). Both WT and MUT mRNAs microinjections resulted in a significant increase in *myog* expression at 24 hpf (Fig. 5b).

We also examined the relative expression of muscle-specific transcripts, including *mylpfa* (*myosin light chain*,* phosphorylatable*,* fast skeletal muscle a)* and *smyhc1* (a myosin specifically involved in slow-twitch skeletal muscle fiber contraction). At 24 hpf, *mylpfa* expression was reduced in both WT and MUT microinjected embryos, while at 48 hpf, its expression was increased in WT samples, as was that of *smyhc1* (Fig. 5b), suggesting that overexpression of both normal and mutated *TNPO3* affects muscle gene expression in a pattern similar to that observed in C2C12 cells.

As for the C2C12 cell model, we investigated the expression of myomiRs, that are highly conserved across species, including zebrafish [51], due to their role in regulating key developmental processes. qRT-PCR analysis showed that at 24 hpf, miR-206, miR-1, and miR-133a (but not miR-133b) were upregulated in embryos microinjected with h*TNPO3* MUT mRNA, while all four myomiRs were downregulated (statistically significant for miR-206 compared to NI embryos) following h*TNPO3* WT mRNA microinjection (Fig. 5c). At 48 hpf, miR-206 and miR-1 levels decreased, while miR-133a and miR-133b increased in MUT microinjected embryos compared to NI and WT microinjected embryos. In contrast, WT microinjected embryos showed myomiRs levels similar to NI, except for the miR-133b downregulation.

### Evaluation of LGMDD2 specific genes and proteins expression in zebrafish

**Fig. 6:**
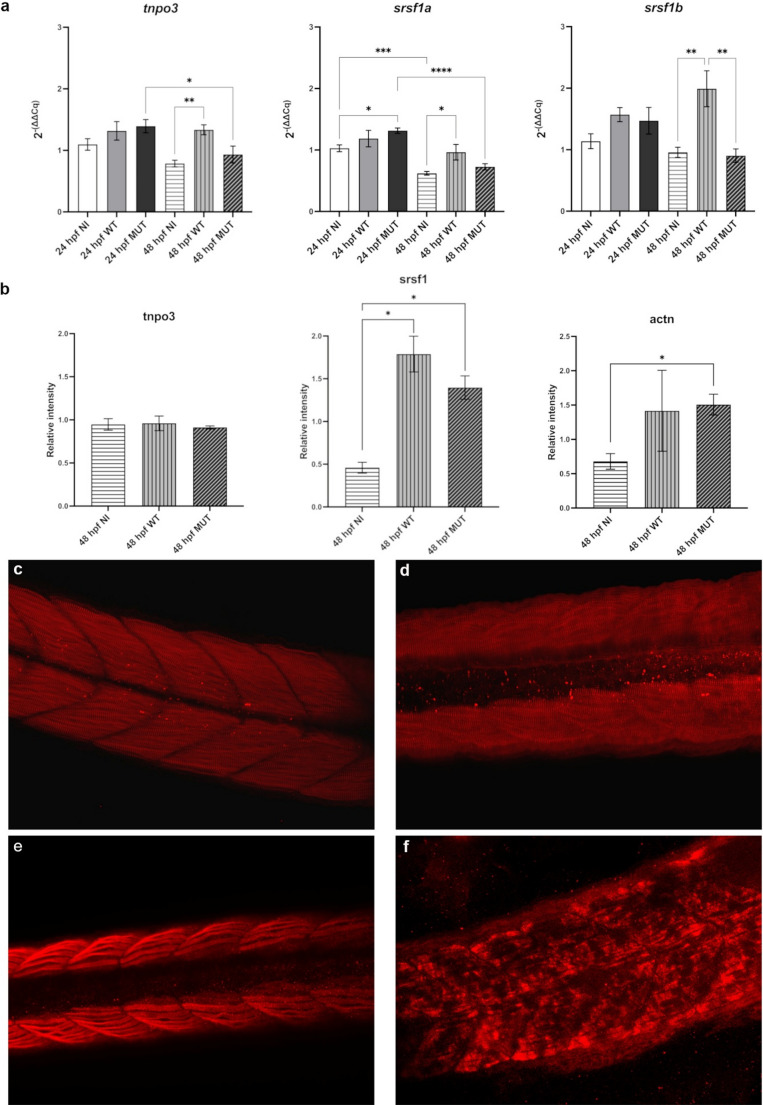
(**a**) Graphical representation of relative gene expression levels of *tnpo3*,* srsf1a*,* srsf1b* at 24 hpf and 48 hpf. (**b**) Western blot analysis for tnpo3, srsf1, and α-actinin at 48 hpf. Graphs express protein quantities normalized to ACTB. Statistical analysis was performed using one-way Anova, Bonferroni’s Multiple Comparison test (*n* ≥ 3); *p < 0,05; **p < 0.01; ***p < 0.001; ****p < 0.0001. Statistical significance refers to comparison among NI, WT and MUT groups at each developmental stage (24 or 48 hpf). NI are non-injected embryos; WT and MUT refer to embryos microinjected with h*TNPO3* WT and MUT mRNA, respectively. IF staining in the caudal portion of zebrafish embryos for α-actinin in (**c**) NI embryos and (**d**) embryos microinjected with *TNPO3* MUT mRNA, and for myosin in (**e**) NI embryos and (**f**) embryos microinjected with *TNPO3* MUT mRNA, all at 48 hpf

Endogenous *tnpo3* expression increased at 24 hpf in both WT and MUT microinjected samples. At 48 hpf, it remains elevated in WT, while it decreases significantly in MUT embryos compared to the same samples at 24 hpf (Fig. 6a).

We also evaluated the expression of the splicing factor *srsf1*, which is a duplicated gene in zebrafish resulting in two different transcripts both annotated as human orthologs: *srsf1a* and *srsf1b*. s*rsf1a* showed a general upregulation, except for a decrease in h*TNPO3* MUT samples at 48 hpf. s*rsf1b* was significantly upregulated in WT and downregulated in MUT embryos at 48 hpf, suggesting that both *TNPO3* overexpression and mutation influence *srsf1* expression during muscle differentiation in zebrafish (Fig. 6a).

Protein analysis at 48 hpf confirmed an increasing expression of srsf1 in both WT and MUT microinjected embryos. α-actinin, a cytoskeletal protein important for muscle development, was significantly increased in MUT embryos compared to NI samples (Fig. 6b). To verify the effect of h*TNPO3* MUT mRNA on muscle structure organization, we analysed ZF embryos at 48 hpf. Immunofluorescence for α-actinin and myosin on the caudal portion in NI samples revealed a normal sarcomere organization with parallel myofibrils and a distinct V-shaped myoseptum (Fig. 6c and e). In contrast, MUT microinjected larvae showed a poorly defined V-shaped myoseptum and mild myofibrillar disarray with α-actinin (Fig. 6d) and an irregular, mosaic fashion pattern for myosin (Fig. 6f).

**Fig. 7:**
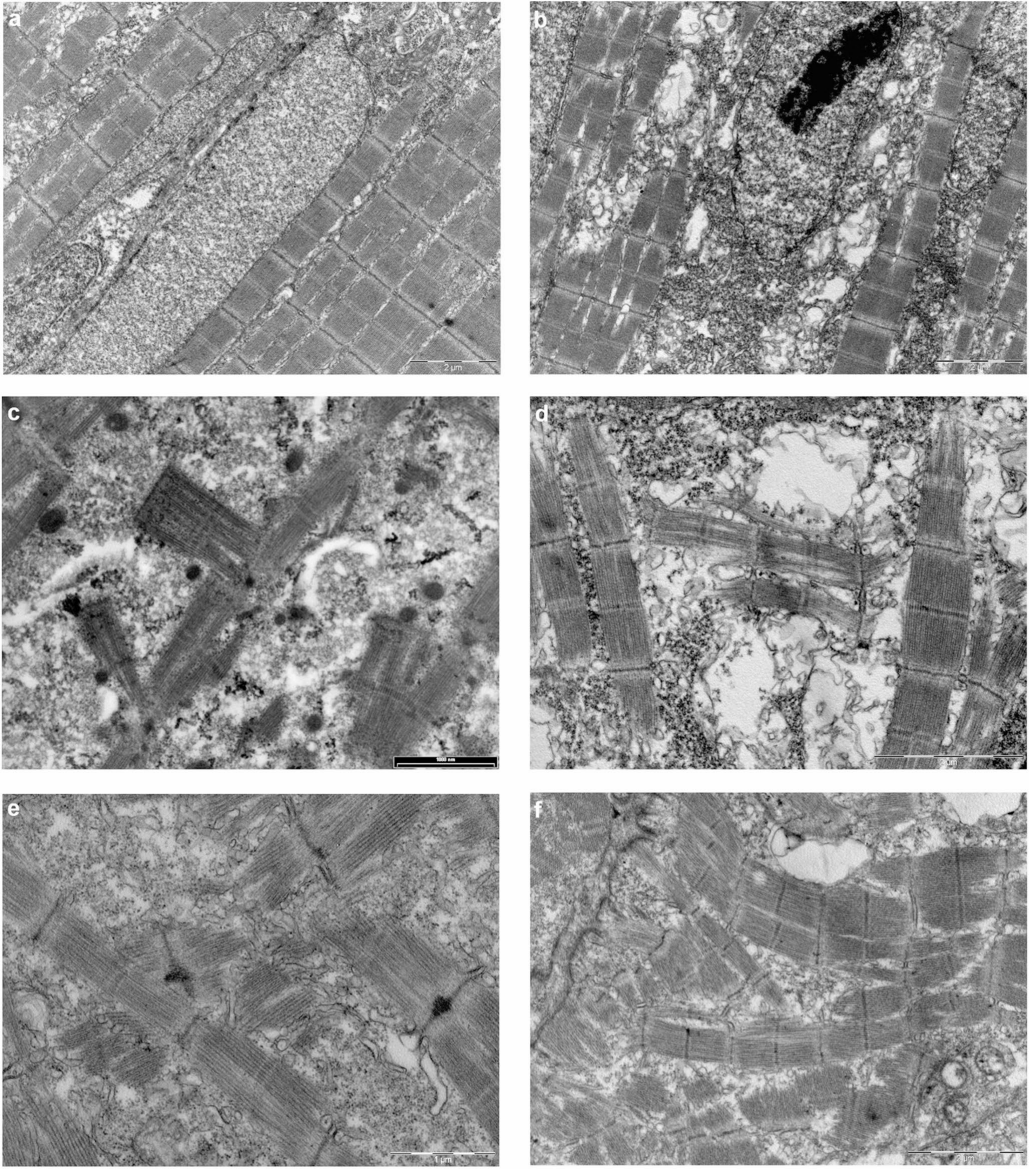
TEM images of NI embryos (**a**, 13500X) and embryos microinjected with *TNPO3* MUT mRNA (**b**, 13500X; **d**, 34000X) both at 48 hpf. TEM images on skeletal muscle biopsies of LGMDD2 patients showing marked myofibrillar disarray (**c**, 34000X). Embryos microinjected with *TNPO3* MUT mRNA at 4 dpf (e at 34000x and f at 25000X)

Ultrastructural characterization of ZF embryos by TEM revealed normal myofiber architecture in NI samples, with parallel aligned myofibrils and nuclei with dispersed chromatin, suggesting an active transcription typically seen at 48 hpf (Fig. 7a). In contrast, embryos microinjected with h*TNPO3* MUT showed disarray in the myofibrillar network, with fibers oriented perpendicularly to the axis of adjacent ones (Fig. 7d), resembling the architectural disarray observed in LGMDD2 patients (Fig. 7c) [6]. Additionally, these embryos showed several nuclei with prominent nucleolus, a feature typical of transcriptionally inactive cells in a more quiescent phase (Fig. 7b). Myofibrillar disarray persisted in embryos at 4 dpf, with myofibrils perpendicularly (Fig. 7e) or haphazardly oriented (Fig. 7f), while no myofibrillar or nuclear alterations were observed nor in NI embyos (Online resource 4) nor in embryos microinjected with *hTNPO3* WT (Online Resource 5). This architectural alteration is specific of embryos microinjected with *hTNPO3* MUT and kept during zebrafish development, confirming the specific effect of the mutated transcript on muscle ultrastructure.

### Functional behavioural analyses

**Fig. 8:**
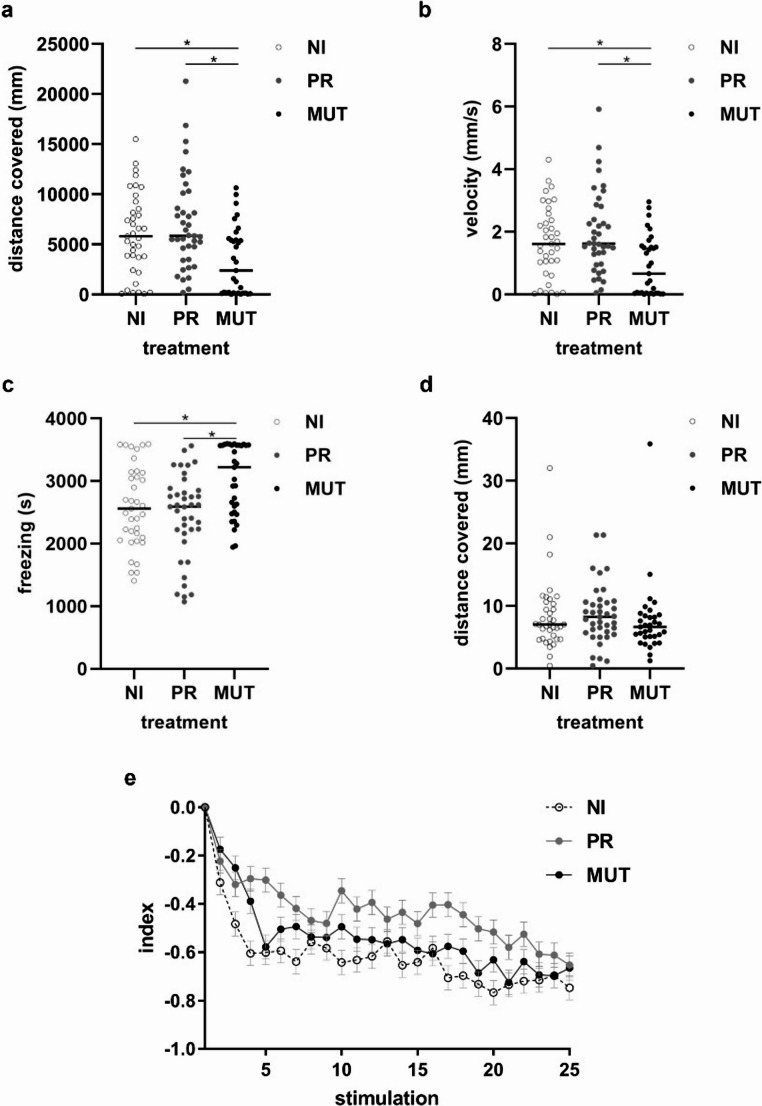
Graphical representation of functional behavioural analyses assayed via the open-field test (a, b, c) and habituation learning test (d, e). (**a**) distance covered by larvae; (**b**) swimming velocity; (**c**) freezing behaviour; (**d**) startle response to the first stimulation; (**e**) habituation learning index. NI: non microinjected embryos; PR: embryos microinjected with phenol red; MUT: embryos microinjected with MUT *hTNPO3* mRNA; *p < 0.05

Overall, we observed a reduction in activity on MUT larvae compared to both controls. Indeed, MUT subjects significantly covered less distance (Fig. 8a), swam at slower velocities (Fig. 8b) and exhibited increased freezing behaviour (Fig. 8c). As expected, the NI and phenol red (PR) groups displayed similar behavioural responses to the novel environment.

In the habituation learning test, larvae did not differ in their startle response to the first mechanical stimulation (F_2,106_ = 0.391, *P* = 0.678; Fig. 8d). The total distance covered by the larvae in response to the 25-repeated stimulations also did not differ between groups (F_2,106_ = 1.089, *P* = 0.340). As expected, the habituation index decreased with repeated stimuli (F_1,2613_ = 303.977, *P* < 0.001; Fig. 8e), but this reduction was consistent across all groups (interaction time * treatment: F_2,2613_ = 1.013, *P* = 0.363), suggesting no treatment-related effect on learning ability. There were no significant differences in individuals’ size among groups (MUT = 2.92 ± 0.34 mm; PR = 3.07 ± 0.28 mm; NI = 3.00 ± 0.30 mm; One-way Anova: F_2,106_ = 2.245, *P* = 0.111).

## Discussion

LGMDD2 is a rare autosomal dominant neuromuscular disorder caused by a heterozygous mutation in the *TNPO3* gene, which encodes a longer protein in its C-terminal domain [7, 8, 12]. TNPO3 normally binds SR-proteins for their nuclear import. Although it is ubiquitously expressed and its role in the nuclear transport of splicing-related proteins is well established [1], its function in skeletal muscle development and its contribution to LGMDD2 pathogenesis remain under investigation. To address this, we developed both in vitro and in vivo models to investigate the role of TNPO3 during myogenesis and in the pathogenetic mechanism of LGMDD2.

As an in vitro model, we used the C2C12 cell line transfected with plasmids encoding the human WT or MUT *TNPO3* sequence. Since C2C12 are usually employed for in vitro muscle diseases modeling [53], transfection with the mutated *TNPO3* was performed to reproduce the LGMDD2 condition, mimicking the genetic background of patients in which both wild-type and mutated forms of *TNPO3* coexist [8]. Transfection with h*TNPO3* WT also allowed us to study the effects of *TNPO3* overexpression in skeletal muscle. For the in vivo model, we used zebrafish due to the developmental and anatomical similarities of skeletal muscle with the human one, as well as the high conservation of myogenic pathways [54] and the significant sequence homology between zebrafish tnpo3 and its human ortholog. Similarly to the cell model, mRNAs encoding WT or MUT human *TNPO3* were microinjected into zebrafish embryos. In both models, we analysed the expression of myogenesis-related genes, such as MRFs, muscle-specific proteins and proteins related to LGMDD2. In addition, we evaluated the expression of myomiRs, due to their interaction with MRFs in regulating the myogenic process. Interestingly, expression of both WT and MUT h*TNPO3* significantly altered the expression of key MRFs. In particular, we noticed that in C2C12 cells, *Myf5* expression remained elevated throughout differentiation, while *MyoG* was consistently downregulated. Similarly, in zebrafish, *myf5* and *myod1* were upregulated in microinjected embryos, with a notably increase in *myog* expression at 24 hpf. These trends are consistent with known regulatory hierarchies: *Myf5* is the first activated MRF and appears to recruit MyoD at an early stage of myogenic commitment and myogenic precursors proliferation. In contrast, *MyoG* support the later stages of myogenic differentiation [55]. The early elevated expression of *Myf5* and *MyoD*, in both in vitro and in vivo models expressing the mutated form of TNPO3, suggests a boosted onset of muscle maturation. Notably, all samples expressing the h*TNPO3* WT also showed expression changes similar to those observed in h*TNPO3* MUT samples. These findings require further investigation, although they could be attributed to *TNPO3* overexpression at the transcriptional level, as its endogenous expression is maintained during C2C12 differentiation and zebrafish development. The contemporary dysregulation of *Myog* in both models suggests that compensatory mechanisms may be activated to promote terminal differentiation in response to increased early MRF activation. MRFs are also involved in MEF2 family protein cross-regulation and regulatory mechanisms, both working in coordination in muscle-specific gene expression [24, 56]. Dysregulation of MEF2c isoforms has been identified in muscle disorders that resemble in some traits LGMDD2, such as myotonic dystrophies (DM1 and DM2) [57]. Moreover, SRSF1, along with other TNPO3 cargoes, has been involved in the regulation of *Mef2c* splicing [2, 24], reinforcing the relationship between TNPO3 and myogenic developmental processes.

Our findings suggest a complex interplay between TNPO3 and the myogenic regulatory network. The presence of mutated TNPO3 induces abnormal gene expression patterns of MRFs and this effect, particularly on the timing and regulation of key MRFs, potentially impairs muscle development and contributes to LGMDD2 pathogenesis.

The regulatory role of myomiRs in myogenesis has been reported in previous studies [27, 28], but no studies have investigated the role of myomiRs in LGMDD2 using zebrafish model. Interestingly, the upregulation of all myomiRs in C2C12 cells transfected with h*TNPO3* WT in the early stages of differentiation (T0-T1 and T5) suggests a positive role of TNPO3 in coordinating the expression of MRFs and MEF2 [33, 34]. Previous reports indicate that MyoG upregulates miR-206 [58] and that miR-1 and miR-206 can reduce Myf5 and MyoG levels [59], which aligns with our observations of decreased MRF expression in late myogenic phase as myomiR levels rise. An additional noteworthy aspect of these results in WT and MUT transfected C2C12 is the upregulation of miR-133a and miR-133b, whose known functions concern the inhibition of muscle differentiation and the promotion of myoblast proliferation [29]. In fact, high levels of miR-133a and miR-133b tend to mantain muscle precursor cells in a less differentiated, proliferative state. Additionally, we observed that miR-133a and miR-133b were downregulated in C2C12 cells transfected with MUT h*TNPO3* compared to WT h*TNPO3* transfected cells. This trend could underline that WT TNPO3 might be involved in a pathway that either promotes the transcription of miR-133 genes or ensures the proper maturation of their precursors. Conversely, the impaired function of the mutant TNPO3 could lead to a disruption of this pathway, resulting in lower levels of mature miR-133a and miR-133b in LGMDD2, and potentially reflecting a cellular response to the disease. In other words, the reduction in miR-133a and miR-133b levels might be a compensatory mechanism to promote muscle repair, even if this cellular attempt is ultimately inefficient or misguided due to the primary TNPO3 defect. In this scenario, miR-133a and miR-133b levels could be proposed as indicators and potential contributing factors to the pathogenetic cascade in correlation with other investigated myomiRs.

Moreover, these data suggest that the presence of mutated TNPO3 could contribute to alter the differentiation during early phases of myogenesis. Elevated myomiR levels in WT samples could be attributed to an excess of WT TNPO3, potentially suppressing early myogenic events. This hypothesis is further supported by the increased expression of other myomiRs, particularly miR-1 and miR-206, which are well known to promote differentiation [27]. On the other hand, analysis at T10 showed a maintenance of this positive regulation with significant values of miR-206 and elevated miR-1 levels. This global trend suggests a possible compensatory mechanism, in which myomiRs associated with promoting differentiation are upregulated to restore correct myogenesis. This mutation-induced compensatory mechanism could modulate myomiR gene expression levels and their interaction results in an altered expression of muscle-specific genes or proteins.

The dynamic expression of MyoG, Myf5, miR-206, and miR-1 in TNPO3 zebrafish model (Fig. 5), with initial upregulation followed by downregulation, may reflect the different phases of a pathological muscle response: an early, acute regenerative phase and a later, chronic-degenerative phase. In particular, at 24 h, the upregulation of MyoG and Myf5 in MUT *hTNPO3* zebrafish model could represent the initiation of a regenerative mechanism. In fact, in response to muscle damage caused by the mutated TNPO3, the MRFs expression begins muscle repair process. Simultaneously, the upregulation of miR-206 and miR-1 by MRFs serves the promoting differentiation. In turn, miR-206 and miR-1 could act in a negative feedback loop by targeting differentiation inhibitors, such as Pax7, ensuring that myoblasts commit to becoming new muscle fibers rather than remaining in a proliferative state [60]. The positive feedback loop (MyoG → miR-206/miR-1) and negative feedback loop (miR-206/miR-1 → Myf5/MyoG) are crucial for tightly controlling the timing of muscle repair. Moreover, the transient upregulation is a hallmark of an acute muscle injury or a compensatory response to chronic damage, where the muscle can be actively trying to repair itself. After 48 h, the downregulation of MyoG, miR-206, and miR-1 is observed in MUT *hTNPO3* zebrafish relative to NI samples, suggesting that the regenerative attempt is failing or is no longer the dominant process. This biphasic expression pattern can reflect the transition from a regenerative phase to a degenerative and fibrotic phase. These findings indicate that the TNPO3 mutation disrupts the temporal regulation of myomiRs, contributing to abnormal muscle maturation. In the context of TNPO3-related muscular dystrophies, such as LGMDD2, and in DM1, previous studies [61, 62] have shown altered levels of miR-206 and miR-1 in patient sera and muscle tissues. These findings support our hypothesis: dysregulation of MRFs or their downstream miRNAs may contribute to impaired muscle regeneration and differentiation observed in these conditions. For instance, elevated miR-206 in DM1 serum (and in our MUT *hTNPO3* model) may represent a compensatory response to muscle damage, while reduced MyoG expression could impair proper miRNA-mediated feedback, exacerbating disease progression. These results emphasize the highly complex regulation of myogenesis supported also by TNPO3 and, more generally, the critical need for a tight balance between myogenic-activating and myogenic-repressing pathways.

To further detail the effect of mutated TNPO3 on the relative expression of muscle-specific proteins during muscle differentiation, we analysed fast and slow myosins (*mylpfa* and *smyhc1*) and *Desmin* in both zebrafish and cell models. In h*TNPO3* WT microinjected zebrafish, it was observed a significant alteration of the *smyhc1* gene expression, particularly at 48 hpf, that is essential for early slow muscle contraction [63] and the reduction of *mylpfa* transcript 24 hpf, whose levels were restored at 48 hpf. Similarly, a significant reduction in *Desmin* transcript was observed in h*TNPO3* transfected C2C12 cells during early differentiation, supporting the hypothesis that both h*TNPO3* WT overexpression and h*TNPO3* MUT presence impair MRF-mediated transcriptional regulation during muscle commitment.

Moreover, we examined the endogenous gene expression levels of *TNPO3*. In C2C12 cells, we noticed a significant increase in *Tnpo3* mRNA during myoblast differentiation, although protein levels remained unchanged. A similar increased trend was observed in zebrafish for both h*TNPO3* WT and MUT microinjected samples. Although the transfection or microinjection of the h*TNPO3* sequence does not alter TNPO3 protein expression, it seems to affect the splicing factor SRSF1 protein level. Our data confirm a dynamic interaction between TNPO3 and SRSF1 during myogenesis and muscle maturation, as previously reported [14].

We also investigated the effects of the h*TNPO3* sequence at morphological level in our zebrafish model, focusing on the cytoskeletal and myofibrillar network, which are altered in LGMDD2 muscle biopsies [6, 12, 64]. Sarcomeric α-actinin, a major component of the Z-disk, normally crosslinks actin and other structural proteins, to maintain an ordered myofibrillar array [65]. In LGMDD2 muscle biopsies, α-actinin exhibits diffuse staining without an organized pattern [17]. Similarly, in our zebrafish model, α-actinin showed a mild disorganization of the myofibrillar network, and the mosaic patter of myosin proves the effect of TNPO3 mutation on myofibrillar differentiation and organization [66, 67]. This myofibrillar disarray was further confirmed at ultrastructural level, as TEM analyses revealed that only embryos microinjected with h*TNPO3* MUT mRNA presented myofibrillar disarray, with myofibrils running perpendicular to adjacent fibers. This feature specifically retraces the alterations observed in LGMDD2 patients muscle biopsies [6].

Another distinct trait of LGMDD2 is autophagy activation accompanied by marked atrophy [6, 12]. To explore this, in the C2C12 model we investigated the expression of *p62* and *MuRF-1*, markers of autophagy and atrophy, respectively. In accordance with histopathological findings from patient muscle biopsies [6], we noticed an increase in *p62* expression during differentiation in cells transfected with either WT or MUT h*TNPO3*. In contrast, *MuRF-1* expression was reduced, despite its usual upregulation under atrophic conditions. This unexpected result could be explained through its regulatory interaction networks involving *Myog*, a gene product known to modulate MuRF-1 and found to be dysregulated in C2C12 cells expressing both WT and MUT h*TNPO3* [68]. Similar changes in *MuRF-1* expression, were described in a previous study using C2C12 cells to model a variant of LGMD caused by a Caveolin 3 mutation [69].

Molecular and morphological investigations in the zebrafish model were followed by functional behavioural studies, showing that MUT larvae exhibited behavioural, but not cognitive, alterations compared to the controls (NI and PR microinjected embryos). When exposed to a novel environment, MUT larvae showed a general reduction in swimming activity, i.e. slower swimming velocity and increased freezing time. This behavioural outcome was expected, since the progressive muscle degeneration observed both in vitro and in vivo, reasonably implied a motor impairment in MUT larvae. However, their ability to learn a simple association task appears unaffected, as MUT larvae displayed escape responses to repeated stimulation comparable to those of the controls. This result aligns with expectations, since the mutation specifically affects skeletal muscle and does not seem to impair the nervous system.

In summary, in both the established models we describe for the first time the involvement of TNPO3 in regulating myogenesis at cellular and organism levels, a role supported by both morphological and behavioural evidence. Furthermore, this study represents a significant step forward in understanding the pathogenic mechanisms of LGMDD2. Our data strongly suggest that the TNPO3 mutation is the underlying cause of the disease onset, interfering with myogenesis regulatory pathways and leading to altered expression of muscle-specific proteins. These models also offer a valuable platform for specific drug testing and repurposing approaches, opening new perspectives for identifying effective therapies for LGMDD2.

## Supplementary Information

Below is the link to the electronic supplementary material.

Supplementary Material 1 (DOCX 16.8 KB) 

Supplementary Material 2 (DOCX 14.9 KB) 

Supplementary Material 3 (DOCX 21.3 KB) 

Supplementary Material 4 (DOCX 570 KB) 

Supplementary Material 5 (DOCX 3.72 MB) 

## Data Availability

Data will be made available from authors on request.
