# Supplementary material for: Novel insights into the molecular mechanisms of LGMDD2: role of TNPO3 in experimental cell and zebrafish models
Source: Cell Mol Life Sci. 2025 Nov 26;82(1):424. doi: 10.1007/s00018-025-05954-9 (PMC12647454; doi:10.1007/s00018-025-05954-9)
Supplement: Supplementary file 1 — Supplementary Material 1 (DOCX 16.8 KB) [file 18_2025_5954_MOESM1_ESM.docx]

**Table 1.** List of primers used for gene expression analyses on C2C12 cells.

| **Gene**  **(GenBank Accession Number)**  **(*Mus musculus*)** | **Primer sequences** |
| --- | --- |
|  |  |
| *Myf5*  *Myogenic Factor 5*  (NM_008656.5) | Fw: 5'-AGGTGGAGAACTATTACAGC |
|  | Rev: 5'-TGATACATCAGGACAGTAGATG |
| *Myog*  *Myogenin*  (NM_031189.2) | Fw: 5'-AGTACATTGAGCGCCTAC |
|  | Rev: 5'-CAAATGATCTCCTGGGTTG |
| *Des*  *Desmin*  (NM_010043.2) | Fw: 5'-ACACCTAAAGGATGAGATGG |
|  | Rev: 5'-GAGAAGGTCTGGATAGGAAG |
| *Murf-1(TRIM63)*  *Muscle-specific RING finger protein 1*  (NM_001039048.2) | Fw: 5'-GACTTAGAACACATAGCAGAG |
|  | Rev: 5'-CTCTTCTGTAAACTCCTCCTC |
| *Sqstm1/p62*  *Sequestosome 1*  (NM_011018.3) | Fw: 5'-AGATCCCAATGTCAATTTCC |
|  | Rev: 5'-CTGAGTGTTACTCTTGTCTTC |
| *Mef2c isoforma α1*  *Myocyte Enhancer Factor 2 C*  (NM_001170537.1) | Fw: 5'-CTCAGACATTGTGGAGACATT |
|  | Rev: 5'-TCAGGGCTGTGACCTACTG |
| *Mef2c isoforma α2*  *Myocyte Enhancer Factor 2 C*  (NM_001347568.1) | Fw: 5'-CTCAGACATTGTGGAGGCAT |
|  | Rev: 5'-TTCTTCAGTGCGTGGGGT |
| *Tnpo3*  *Transportin 3*  (NM_012470.4) | Fw: 5'-GCCAAAGTCTACGGACGG |
|  | Rev: 5'-GACCACAAGCGAGACGTACA |
| *Srsf1*  *Serine and arginine rich splicing factor1*  (NM_006924.5) | Fw: 5'-AGTGGTTGTCTCTGGACTGC |
|  | Rev: 5'-CGTACAAACTCCACGACACC |
| *Gapdh*  *Glyceraldehyde-3-Phosphate*  *Dehydrogenase*  (NM_001256799.2) | Fw: 5'-CTCTGATTTGGTCGTATTGG |
|  | Rev: 5'-GTAAACCATGTAGTTGAGGTC |
