# Supplementary material for: Novel insights into the molecular mechanisms of LGMDD2: role of TNPO3 in experimental cell and zebrafish models
Source: Cell Mol Life Sci. 2025 Nov 26;82(1):424. doi: 10.1007/s00018-025-05954-9 (PMC12647454; doi:10.1007/s00018-025-05954-9)
Supplement: Supplementary file 2 — Supplementary Material 2 (DOCX 14.9 KB) [file 18_2025_5954_MOESM2_ESM.docx]

**Table 2**. List of primary antibodies used for WB and IF analyses.

| Primary Antibodies | Dilution | Company |
| --- | --- | --- |
| Skeletal Muscle Myosin (F59) | For WB: 1/200  For IF: 1/200 | Santa Cruz Biotechnology, Dallas, Texas, USA |
| MyoD (G-1) | For WB: 1/200 |  |
| TNPO3 N-terminal (ab71388)  TNPO3 C-terminal (EPR5264) | For WB: 1/1 000  For IF: 1/200 | Abcam, Cambridge, UK |
| SRSF1 (96) | For WB: 1/250  For IF: 1/100 | Thermo Fisher Scientific, Waltham, Massachusetts, USA |
| Alpha actinin | For WB: 1/500  For IF: 1/500 | Sigma-Aldrich |
| Actin (I-19) | For WB:1/500 | Santa Cruz Biotechnology, Dallas, Texas, USA |
