# Supplementary material for: Novel insights into the molecular mechanisms of LGMDD2: role of TNPO3 in experimental cell and zebrafish models
Source: Cell Mol Life Sci. 2025 Nov 26;82(1):424. doi: 10.1007/s00018-025-05954-9 (PMC12647454; doi:10.1007/s00018-025-05954-9)
Supplement: Supplementary file 3 — Supplementary Material 3 (DOCX 21.3 KB) [file 18_2025_5954_MOESM3_ESM.docx]

**Table 3**. List of primers for gene expression analyses on Zebrafish.

| **Gene**  **(GenBank Accession Number) (*Danio rerio*)** | **Sequenza coppia di primers** |
| --- | --- |
|  |  |
| *myf5*  *myogenic factor 5*  (NM_131576.1) | Fw: 5'-GCTGCTCAGAGAGCATGGTT |
|  | Rev: 5'-GTGCTAGCATTTTGTGCGTCA |
| *myod1*  *myoblast determination protein 1*  (NM_131262.2)) | Fw: 5'- ATGACACACCAAATGCTGACG |
|  | Rev: 5'- GATCCCTCATGCGGAGAACA |
| *myog*  *myogenin*  (NM_131006.1) | Fw: 5'-TCTGAAGAGGAGCACATTGA |
|  | Rev: 5'-AGCCCTGATCACTAGAGGA |
| *smyhc1*  *slow myosin heavy chain 1*  (NM_001020507.1) | Fw: 5'-TGCCAAGACCATCAGAAATG |
|  | Rev: 5'-CACACCAAAGTGAATTCGGATA |
| *mylpfa*  *myosin light chain, phosphorylatable, fast skeletal muscle a*  (NM_131188.2) | Fw: 5'-CCACTCAGTGCGACAGGTT |
|  | Rev: 5'-AACATTGCCAGCCACATCT |
| *tnpo3*  *transportin 3*  (NM_201887.1) | Fw: 5'-CGGCAGCTAAAGCCATCCAT |
|  | Rev: 5'-GCGCTGTACCTTTGAGCAAG |
| *srsf1a*  *serine and arginine rich splicing factor1a*  (NM_213015.1) | Fw: 5'-CATGAGGGAGAAACTGCGTA |
|  | Rev: 5'-GAGTAGCTTCGGCTACGGTT |
| *srsf1b*  *serine and arginine rich splicing factor1b*  (NM_ 200593.2) | Fw: 5'-GTTCGAGGACCCGAGAGATG |
|  | Rev: 5'-GAGGTCCGTATCTGCCTCTG |
| *slc25a5*  *solute carrier family 25 member 5*  (NM_173247.1) | Fw: 5'-CTGCCCGATCCCAAGAACAC |
|  | Rev: 5'-CTTCCTCCAGCAGTCAATTGTGCC |
| *actb2*  *actin beta 2*  (NM_181601.5) | Fw: 5'-GCAGAAGGAGATCACATCCCTGGC |
|  | Rev: 5'-CATTGCCGTCACCTTCACCGTTC |
