# Supplementary material for: Novel insights into the molecular mechanisms of LGMDD2: role of TNPO3 in experimental cell and zebrafish models
Source: Cell Mol Life Sci. 2025 Nov 26;82(1):424. doi: 10.1007/s00018-025-05954-9 (PMC12647454; doi:10.1007/s00018-025-05954-9)
Supplement: Supplementary file 4 — Supplementary Material 4 (DOCX 570 KB) [file 18_2025_5954_MOESM4_ESM.docx]

**Supplementary Figure 1** Ultrastructure of skeletal muscle of non-microinjected embryos at 4 dpf. (a) Regular sarcomeres (magnification 10500X) and (b) well-preserved myofibrils are wholly seen (magnification 25000X).

**a**


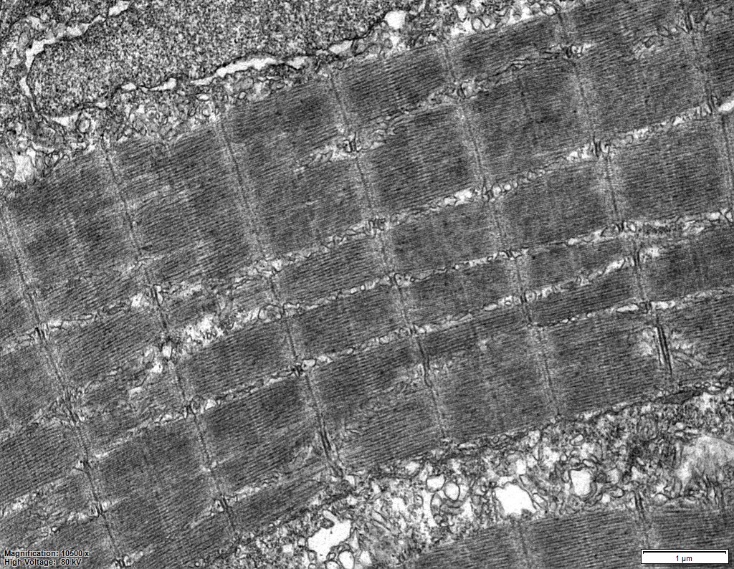


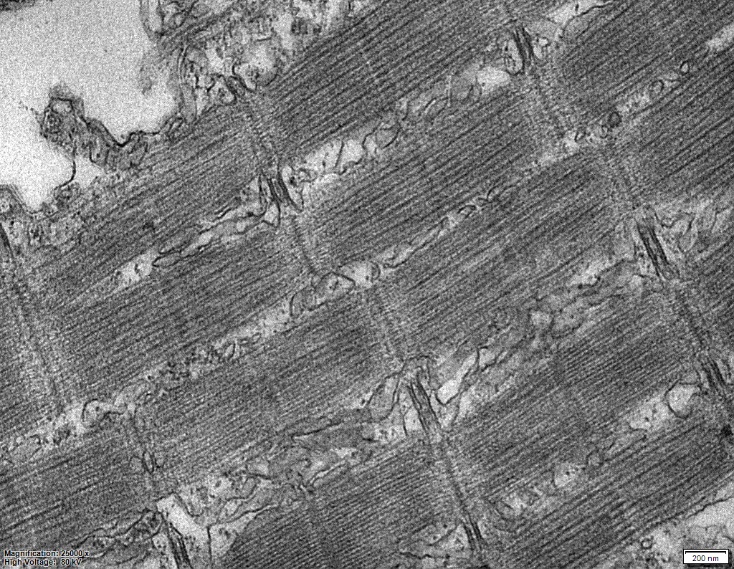


**b**
