# Supplementary material for: Novel insights into the molecular mechanisms of LGMDD2: role of TNPO3 in experimental cell and zebrafish models
Source: Cell Mol Life Sci. 2025 Nov 26;82(1):424. doi: 10.1007/s00018-025-05954-9 (PMC12647454; doi:10.1007/s00018-025-05954-9)
Supplement: Supplementary file 5 — Supplementary Material 5 (DOCX 3.72 MB) [file 18_2025_5954_MOESM5_ESM.docx]

**Supplementary Figure 2** Ultrastructure of embryos microinjected with WT h*TNPO3* mRNA sequence. (a) Nucleus with small nucleolus (arrow), magnification 34000X, (b) myofibrils normally aligned in parallel with intermyofibrillar mitochondria (arrow), magnification 34000X.


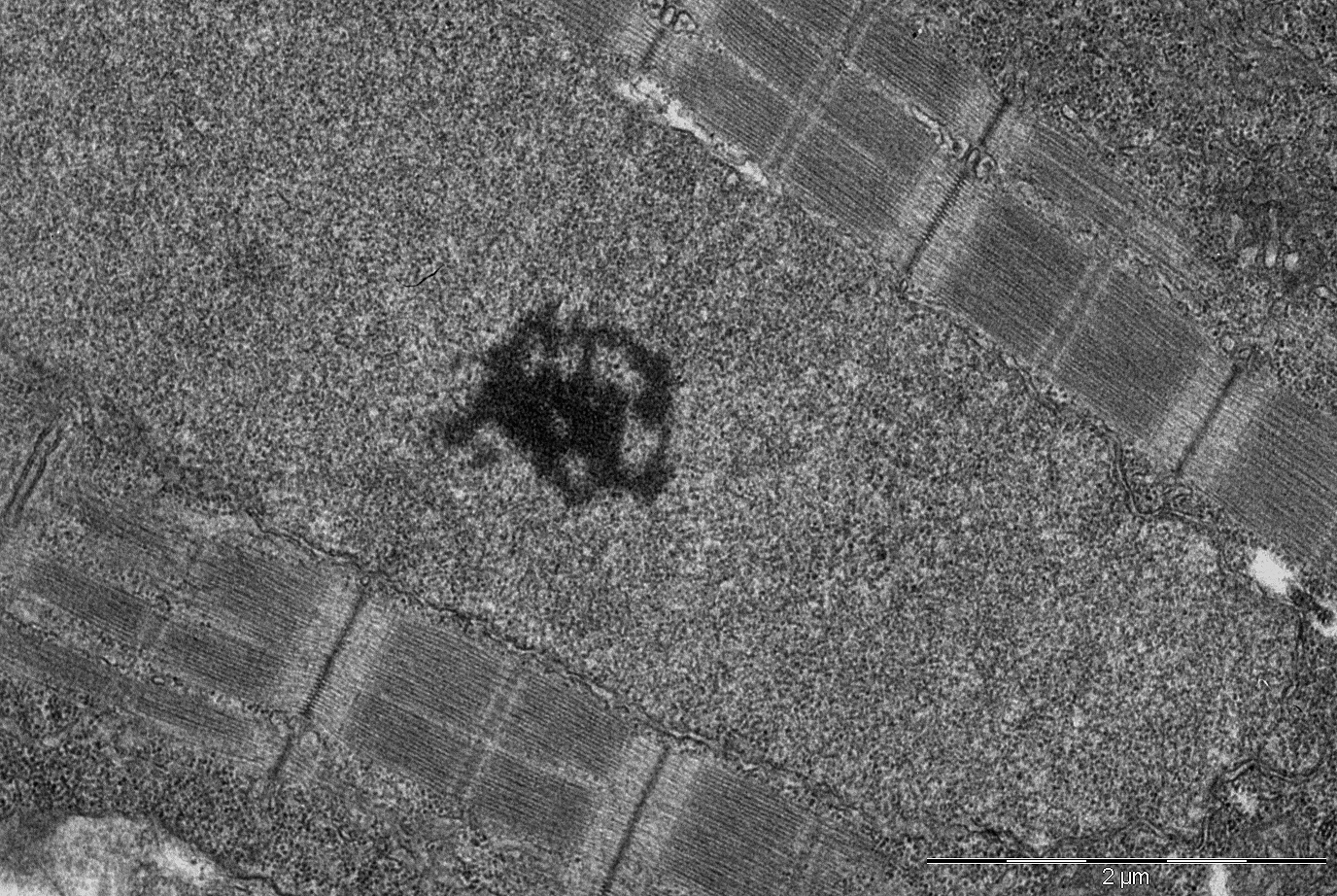


**a**


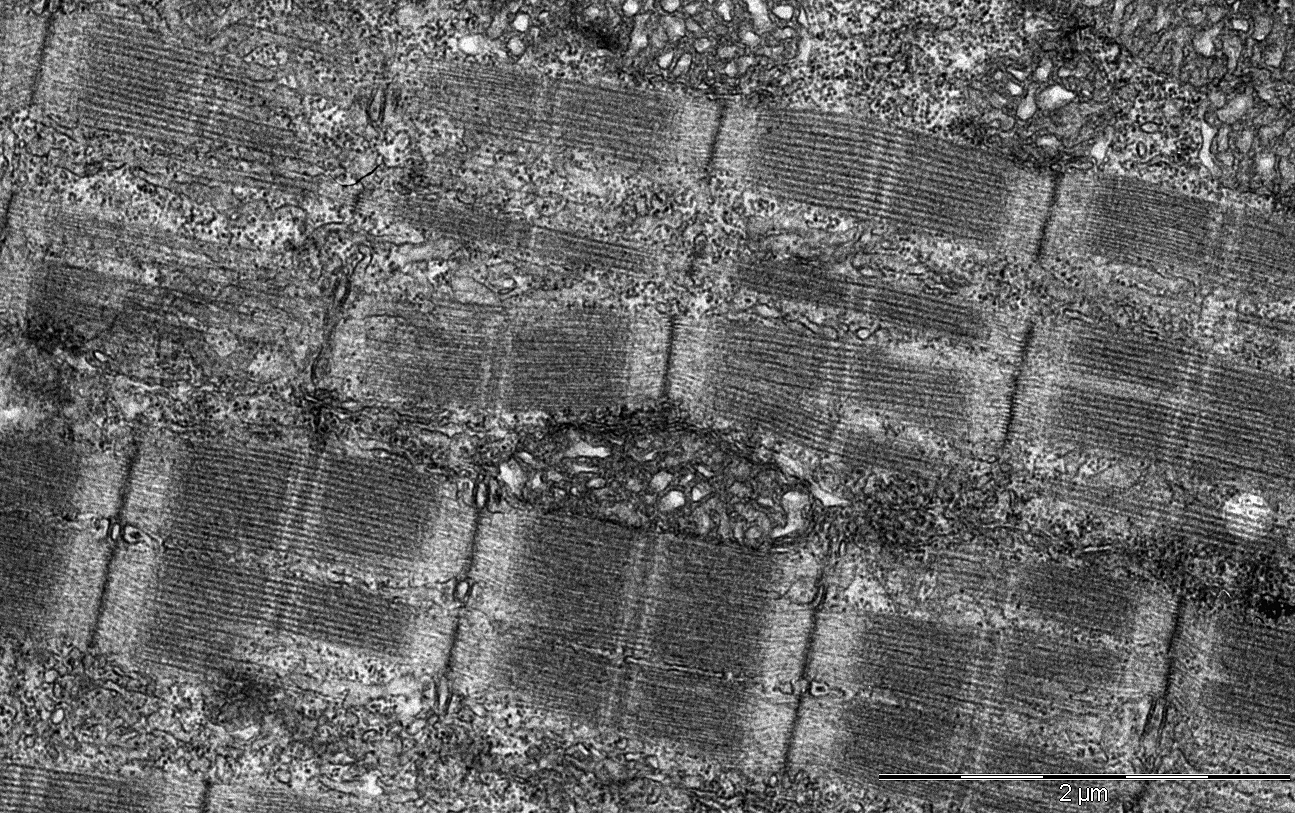


**b**
